# Corneal Arcus, Xanthomas, and Finger Deformities in a Young Woman With Homozygous Familial Hypercholesterolemia

**DOI:** 10.1155/carm/2255274

**Published:** 2025-10-01

**Authors:** Babak Bagheri, Fatemeh Shokri, Amir Hasan Farzaneh

**Affiliations:** ^1^Department of Cardiology, School of Medicine, Cardiovascular Research Center, Mazandaran University of Medical Sciences, Sari, Iran; ^2^Department of Clinical Pharmacy, School of Pharmacy, Mazandaran University of Medical Sciences, Sari, Iran; ^3^Student Research Committee, Faculty of Pharmacy, Mazandaran University of Medical Sciences, Sari, Iran

**Keywords:** case report, familial hypercholesterolemia, PCSK9 inhibitor, xanthoma

## Abstract

**Background:**

Homozygous familial hypercholesterolemia (HoFH) is a rare, autosomal dominant disorder characterized by severely elevated low-density lipoprotein cholesterol (LDL-C), leading to premature cardiovascular disease. Early diagnosis is critical but often delayed due to limited access to genetic testing, particularly in regions with high consanguinity.

**Case Presentation:**

A 23-year-old Persian woman, born to consanguineous parents, presented with tendon and cutaneous xanthomas, bilateral corneal arcus, and hand deformities resembling rheumatoid arthritis. Her medical history included childhood hypercholesterolemia, and her family history revealed premature myocardial infarction in her mother. Laboratory results showed markedly elevated LDL-C (509 mg/dL). Diagnosis was confirmed clinically using Dutch Lipid Clinic Network and Simon Broome criteria, as genetic testing was unavailable.

**Interventions and Outcomes:**

Initial therapy with rosuvastatin (40 mg) and ezetimibe (10 mg) failed to achieve LDL-C targets. Evolocumab (420 mg monthly) was added, resulting in an 82% reduction in LDL-C (from 509 to 89 mg/dL) and partial regression of xanthomas.

**Conclusion:**

This case highlights the diagnostic challenges of HoFH in resource-limited settings and underscores the importance of clinical criteria when genetic testing is inaccessible. Aggressive lipid-lowering therapy, including PCSK9 inhibitors, is essential for managing HoFH and mitigating cardiovascular risk. Early recognition of physical signs (e.g., xanthomas and corneal arcus) and family screening are crucial for timely intervention.

## 1. Introduction

Familial hypercholesterolemia (FH) is a rare, autosomal dominant disorder characterized by markedly elevated low-density lipoprotein cholesterol (LDL-C) from early childhood, leading to premature atherosclerotic cardiovascular disease. The condition has an estimated incidence of 1 in 160–300 individuals worldwide, with increased prevalence in populations where consanguineous marriages are common [1, 2]. FH is caused by three different genetic defects, involving mutations in the LDL receptor gene, APOB, and PCSK9. Most FH patients have LDL receptor gene mutations, which result in elevated LDL cholesterol levels and early-onset atherosclerosis [3].

FH can present in two forms: homozygous (HoFH) and heterozygous (HeFH). The distinction between HoFH and HeFH can be made based on clinical features, LDL-C levels, and genetic testing for confirmation [3].

The American Heart Association (AHA) guidelines define HoFH as having LDL-C levels exceeding 400 mg/dL (10 mmol/L), a parental history of FH in one or both parents, and confirmation through genetic testing. These patients typically show more severe clinical manifestations of premature atherosclerosis and may present with symptoms at an early age. In comparison, HeFH presents with milder LDL-C elevations ranging from 160 to 190 mg/dL, along with a family history of either FH or premature coronary artery disease in a first-degree relative and positive genetic test results. HoFH usually results from mutations affecting both alleles of genes involved in LDL metabolism, most commonly the LDL receptor (LDLR) gene [4].

Patients with HoFH typically present with tendon or cutaneous xanthomas, corneal arcus, and early cardiovascular events such as myocardial infarction or aortic valve disease. Diagnosis relies on clinical features, lipid profiles, family history, and increasingly, genetic testing. Early identification and aggressive lipid-lowering treatment are essential to prevent premature morbidity and mortality [5, 6].

Here, we report a case of HoFH in a 23-year-old Persian woman presenting with classic clinical findings, a strong family history of premature myocardial infarction, and consanguineous parentage.

Despite the availability of numerous case studies and clinical guidelines on FH, the condition often goes unrecognized, particularly in Persian populations. Limited access to diagnostic facilities and genetic testing centers contributes to frequent underdiagnosis. Additionally, the lack of systematic documentation in Persian communities obscures the true prevalence of FH. This case report not only presents a confirmed FH case from Persian regions but also highlights the critical role of clinical evaluation—including physical signs and symptoms—in diagnosing FH when genetic testing is unavailable.

## 2. Case Presentation

A 23-year-old Persian woman was referred to our hospital for evaluation of multiple painless, progressive swallowed nodules on her fingers and elbows and back pain on exertion for 3 months, as well as bilateral corneal discoloration. She was the only child born of a consanguineous marriage (first cousins). Her first nonspecific symptoms appeared around age 5; however, consistent follow-up for dyslipidemia had been interrupted, with the last visit approximately 2 years prior.

**Figure 1:**
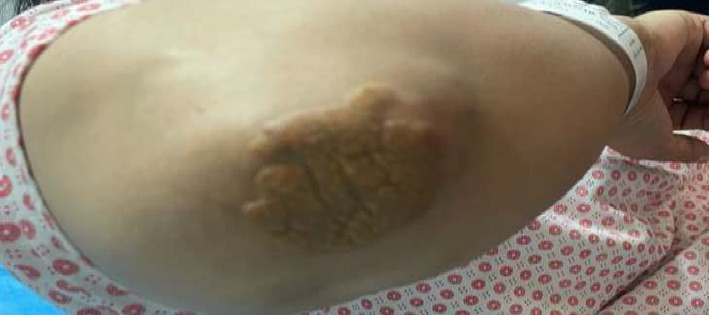
Cutaneous xanthoma (multiple, yellowish papules with an erythematous base) on the external surface of both elbows.

**Figure 2:**
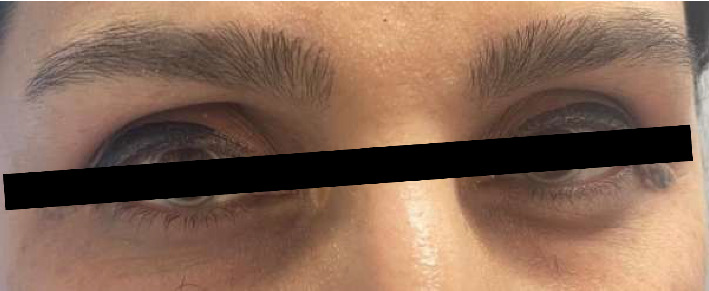
Corneal arcus in both eyes (bilateral grayish-white rings in both eyes are visible along the peripheral corneal margin).

**Figure 3:**
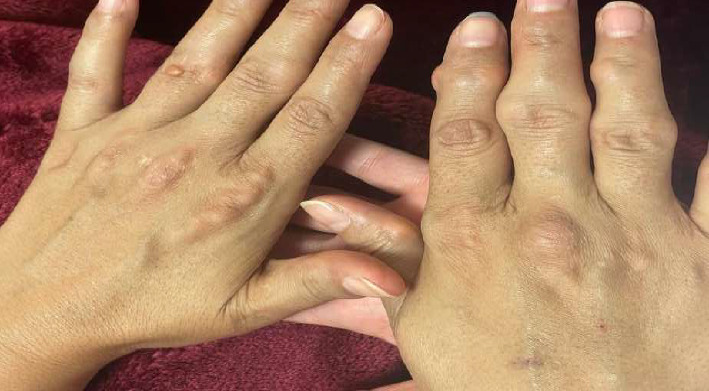
Tendon xanthoma (firm, nodules located in the external surface of both hands) with deformities in both hands.

Physical examination revealed multiple firm, elevated, yellowish nodular plaques, without discomfort, pain, or pruritus, over the extensor surfaces of the elbows consistent with cutaneous xanthomas (Figure 1), and a prominent gray arc along the corneal margins bilaterally (Figure 2). The patient exhibited tendon xanthomata on both hands, primarily affecting the proximal interphalangeal (PIP) joints. These xanthomatous deposits led to deformities of the PIP, distal interphalangeal (DIP) joints, and metacarpophalangeal (MCP) joints, creating a pattern similar to that seen in rheumatoid arthritis. Notably, there were no signs of joint inflammation (Figure 3). Vital signs were stable and within normal limits, and her body mass index was 21 kg/m^2^. Transthoracic echocardiography and electrocardiography (ECG) were unremarkable. Additional cardiovascular risk stratification with carotid ultrasound or coronary calcium scoring was not performed due to limited availability of resources at the time; however, She was referred to a well-equipped center to perform these procedures.

**Figure 4:**
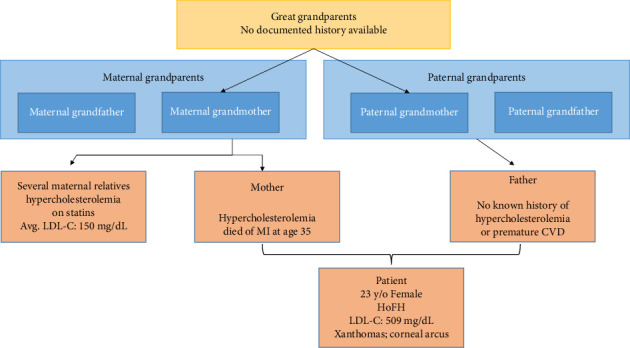
Pedigree chart.

Her medical history was significant for hypercholesterolemia diagnosed in childhood. She denied smoking or alcohol consumption and reported regular exercise with a balanced diet. Family history was remarkable for her mother's sudden death from myocardial infarction at age 35 and widespread hypercholesterolemia among maternal relatives, most of whom were on statin therapy with LDL-C levels averaging 150 mg/dL. A pedigree chart is presented in (Figure 4).

Initial laboratory evaluation revealed total cholesterol (602 mg/dL), LDL-C (509 mg/dL), HDL-C (74 mg/dL), and triglycerides (89 mg/dL), consistent with severe primary dyslipidemia. Liver function, thyroid function, hemoglobin A1c, and albumin levels were normal. Urinalysis and abdominal ultrasonography were unremarkable. All secondary causes of hypercholesterolemia including nephrotic syndrome, hypothyroidism, and drug-induced effects, were ruled out during the diagnostic workup.

Diagnostic scoring with the Dutch Lipid Clinic Network (DLCN) and Simon Broome criteria classified the patient as having “definite familial hypercholesterolemia”. Given her markedly elevated LDL-C, early onset of symptoms, and family history, a diagnosis of HoFH was made.

Despite the clinical suspicion of FH, this patient could not undergo confirmatory genetic testing for key markers—including the LDLR, PCSK9, and apolipoprotein B100 (apoB100) genes—due to limited access. Instead, the diagnosis was established clinically, based on a combination of the patient's medical history, physical findings (e.g., tendon xanthomas), highly elevated lipid levels, and application of standard diagnostic criteria. This highlights the challenges of diagnosing FH in resource-constrained settings where genetic testing remains unavailable.

In addition to the unavailability of genetic testing, another limitation was our inability to assess the cholesterol levels of the patient's mother.

Figure 4 shows that she was the only child born of a consanguineous marriage (first cousins). Family history was remarkable for her mother's sudden death from myocardial infarction at age 35 and widespread hypercholesterolemia among maternal relatives, most of whom were on statin therapy.

## 3. Treatment and Follow-Up

A multidisciplinary treatment approach was implemented, incorporating nutritional adjustments, behavioral lifestyle interventions, and evidence-based pharmacotherapy. The patient had been treated with high-intensity lipid-lowering therapy, including rosuvastatin 40 mg and ezetimibe 10 mg daily, for 2 years. Despite this, her LDL-C levels remained above target, failing to meet the European Society of Cardiology (ESC) recommended goal of < 55 mg/dL or the European Atherosclerosis Society (EAS) target of < 70 mg/dL for very high-risk patients.

Based on presumed residual LDL receptor activity, evolocumab 420 mg monthly, a PCSK9 inhibitor, was initiated. The combination therapy was well tolerated, with no adverse events except mild alopecia deemed unrelated to treatment. Over subsequent months, LDL-C levels decreased substantially, and partial regression of xanthomas was observed (Table 1).

Genetic counseling was provided to the patient and family members, stressing the necessity of screening given the autosomal dominant transmission of FH. Longitudinal follow-up will involve coordinated care with serial lipid assessments and echocardiographic evaluation.

## 4. Discussion

HoFH is a rare but serious genetic disorder leading to early-onset cardiovascular disease. It primarily results from biallelic LDL receptor mutations, though other genetic causes exist. Clinical features often include tendon and cutaneous xanthomas, corneal arcus, and premature myocardial infarction [7].

Our patient's clinical presentation, family history, and lipid profile were consistent with HoFH. The consanguineous background further supports a homozygous or compound heterozygous mutation. Diagnosis was supported by validated clinical criteria, despite lack of genetic confirmation.

This case presented an unusual clinical manifestation characterized by finger deformities resembling rheumatoid arthritis—a rare finding in FH patients. Such atypical presentations may create diagnostic challenges, necessitating thorough differential diagnosis exclusion. These findings underscore the need for clinicians to maintain high clinical suspicion for FH when evaluating patients with uncommon symptoms, especially those with positive family history and premature cardiovascular disease. The patient's widespread xanthomas reflect a strong genetic predisposition. Unlike their rare occurrence in routine clinical settings, tendon xanthomas represent highly specific diagnostic indicators for FH, with differential manifestations across genetic subtypes of heterozygous and homozygous forms [5, 8, 9]. Our patient's early age-onset lesions exemplify the characteristic HoFH phenotype.

The deposition of cholesterol in various tissues drives the development of both cutaneous and tendon xanthomas while also contributing to the pathogenesis of coronary artery disease and aortic valve pathology [10, 11].

Per DLCN guidelines, corneal arcus carries substantial diagnostic value (4 points). Premature arcus strongly correlates with youthful LDL-C elevation, but its link to CAD remains unclear. While Framingham data suggested 4–8 years' predictive value for CVD, this association was non-significant after age/sex adjustment [12, 13].

**Figure 5:**
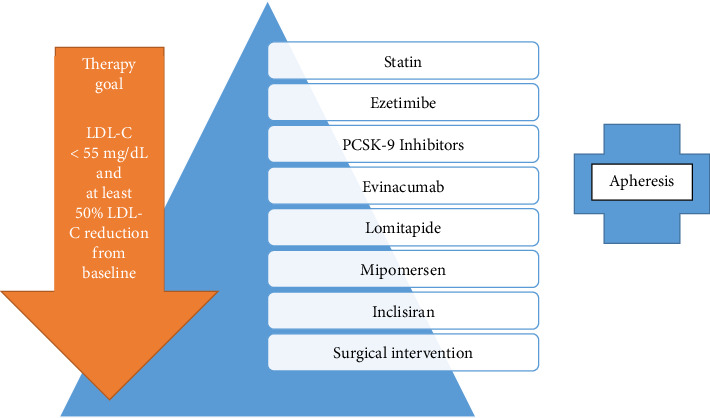
Treatment algorithm for homozygous familial hypercholesterolemia.

Treatment is challenging due to impaired LDL receptor activity, rendering statins and ezetimibe insufficient alone. The addition of PCSK9 inhibitors, such as evolocumab, can improve LDL-C levels significantly in patients with residual receptor function, as seen in this case. Advanced therapies, including lomitapide or LDL apheresis (Figure 5), may be required for some [14–16].

The patient's strong family history and history of cutaneous manifestations prior to diagnosis underscore critical gaps in awareness and healthcare accessibility, particularly in remote regions of Iran. This case emphasizes the vital role of recognizing physical signs—such as xanthomas and corneal arcus—in identifying FH in resource-limited settings where genetic testing and cascade screening are often unavailable. While genetic confirmation remains the gold standard, clinical diagnosis based on phenotypic features is indispensable in such contexts. Early detection of FH and prompt initiation of lifestyle modifications and lipid-lowering therapy are essential to mitigate the risk of premature atherosclerotic complications.

## 5. Conclusion

This case underscores the importance of early diagnosis, family screening, and aggressive lipid management, especially in populations with high consanguinity rates. We report a young Persian woman with HoFH characterized by severe hypercholesterolemia, tendon and cutaneous xanthomas, corneal arcus, and premature maternal myocardial infarction. Despite maximal statin and ezetimibe therapy, LDL-C targets were only approached after adding evolocumab. Early identification and comprehensive treatment are vital to reducing cardiovascular risk in HoFH.

## Figures and Tables

**Table 1:** Lipid profile of the patient.

Time	TC (mg/dL)	LDL-C (mg/dL)	HDL-C (mg/dL)	TG (mg/dL)	Pharmacotherapy
Day 0	602	509	74	89	Rosuvastatin 40 mg daily
					Ezetimibe 10 mg daily
					Asprin 80 mg daily
					Add evolocumab 420 mg monthly

Day 29	499	413	69	82	Rosuvastatin 40 mg daily
					Ezetimibe 10 mg daily
					Change evolocumab 140 mg every 2 weeks

Day 49	266	197	58	56	Continue previous therapies

Day 79	156	89	59	43	Rosvastatin 40 mg daily
					Ezetimibe 10 mg daily (82% LDL-C reduction from baseline)
					Discontinue evolocumab

*Note:* TG, triglyceride.

Abbreviations: HDL-C, high-density lipoprotein cholesterol; LDL-C, low-density lipoprotein cholesterol; TC, total cholesterol.

## Data Availability

The data that support the findings of this study are available on request from the corresponding author. The data are not publicly available due to privacy or ethical restrictions.

## Nomenclature

ApoB100: Apolipoprotein B100
CAD: Coronary artery disease
CVD: Cardiovascular disease
DIP: Distal interphalangeal
DLCN: Dutch Lipid Clinic Network
ECG: Electrocardiogram
FH: Familial hypercholesterolemia
heFH: Heterozygous FH
HoFH: Homozygous familial hypercholesterolemia
LDL: Low-density lipoprotein
LDL-C: Low-density lipoprotein cholesterol
LDLR: Low-density lipoprotein receptor
PCSK9: Proprotein convertase subtilisin/kexin type 9
PIP: Proximal interphalangeal
XP: Xanthelasma palpebrarum
